# Acupuncture for Low Back Pain: An Overview of Systematic Reviews

**DOI:** 10.1155/2015/328196

**Published:** 2015-03-04

**Authors:** Lizhou Liu, Margot Skinner, Suzanne McDonough, Leon Mabire, George David Baxter

**Affiliations:** ^1^Centre for Health, Activity and Rehabilitation Research, School of Physiotherapy, University of Otago, P.O. Box 56, 325 Great King Street, Dunedin 9054, New Zealand; ^2^Centre for Health and Rehabilitation Technologies, Institute of Nursing and Health Research, School of Health Sciences, University of Ulster, Shore Road, Newtownabbey, County Antrim BT37 0QB, UK

## Abstract

*Objective*. As evidence of the effectiveness of acupuncture for low back pain (LBP) is inconsistent, we aimed to critically appraise the evidence from relevant systematic reviews.* Methods*. Systematic reviews of randomized controlled trials (RCTs) concerning acupuncture and LBP were searched in seven databases. Internal validity and external validity of systematic reviews were assessed. Systematic reviews were categorized and high quality reviews assigned greater weightings. Conclusions were generated from a narrative synthesis of the outcomes of subgroup comparisons.* Results*. Sixteen systematic reviews were appraised. Overall, the methodological quality was low and external validity weak. For acute LBP, evidence that acupuncture has a more favorable effect than sham acupuncture in relieving pain was inconsistent; it had a similar effect on improving function. For chronic LBP, evidence consistently demonstrated that acupuncture provides short-term clinically relevant benefits for pain relief and functional improvement compared with no treatment or acupuncture plus another conventional intervention.* Conclusion*. Systematic reviews of variable quality showed that acupuncture, either used in isolation or as an adjunct to conventional therapy, provides short-term improvements in pain and function for chronic LBP. More efforts are needed to improve both internal and external validity of systematic reviews and RCTs in this area.

## 1. Introduction

Low back pain (LBP) which refers to pain and discomfort localized in the lumbosacral region, with or without radiating leg pain [1], is prevalent in the general population [2]. It is estimated that over 70% of adults in industrialized countries suffer from LBP during a lifetime [3]. With growing evidence of high prevalence in developing countries [4–6], LBP is no longer recognized as a disorder confined to high-income nations but is a major health problem globally [7]. The functional limitations and consequent disability create a heavy economic burden on individuals and society: expenditure on LBP in the United States has been estimated to be at least $100 billion per year [8, 9].

Although a considerable variety of conservative therapy alternatives are available for the treatment of LBP, no single modality appears to be superior [2]. Patients are often dissatisfied with conventional medical approaches and turn to complementary and alternative medicines to manage their symptoms [10, 11], among which acupuncture is one of the most popular options [12].

As an ancient medical procedure that is commonly used, acupuncture has gained increasing interest from the public as well as health professions [11, 13]. However, effectiveness of acupuncture for the management of LBP is not without dispute: over the past quarter of a century, numerous systematic reviews have investigated the effectiveness of acupuncture in the management of LBP, but review conclusions are sometimes contradictory and often limited by the quantity and quality of the included studies. In the past decade, three clinical practice guidelines have been published with inconsistent conclusions regarding the recommendations for acupuncture in the treatment of chronic LBP [1, 14, 15]; this has led to confusion for clinicians when making evidence-based clinical decisions concerning acupuncture. Given this, an overview of evidence provided by these reviews would overcome the limitations of the single systematic review, allowing a systematic assessment of the strength of the current evidence, and comprehensive analysis of the results of existing systematic reviews.

Overviews are a relatively new approach to evidence synthesis and have become increasing popular in health care literature [32]; this approach has particular relevance for areas with overlapping reviews. While systematic reviews appear at the top of the “hierarchy of evidence” that informs evidence-based practice, comparative data across a variety of different domains are often lacking (e.g., the combination of different interventions, outcomes, conditions, problems, or populations). However, such data are critical for decision-makers including clinicians, policy makers, and informed consumers [32, 33]. Additionally, for an overview evidence from multiple systematic reviews relevant to a single condition are compiled and consistency of findings is explored across these reviews [34].

The objective of this overview was, therefore, to summarize and critically appraise the evidence of relevant systematic reviews and to present a comprehensive evaluation of the therapeutic value of acupuncture for LBP.

## 2. Methods

### 2.1. Search Strategy

A comprehensive computer-aided literature search was undertaken in the following databases from their inception until February 2014: Medline, EMBASE, AMED, CINAHL, Cochrane Library, and two Chinese databases, the China National Knowledge Infrastructure (CNKI) and the Wan Fang Database that include “grey literature,” such as dissertations and conference reports. Search terms used were (systematic review OR meta-analysis) AND (acupuncture OR acupuncture therapy OR acupuncture points OR needle OR electro-acupuncture OR auricular-acupuncture OR warm-acupuncture OR dry needling OR trigger-point therapy OR moxibustion) AND (low back pain OR back pain OR backache OR lumbago OR sciatica OR dorsalgia) with slight modifications for individual searches in each database. Boolean operators were used and the search was limited to adult participants. Reference lists of all papers retrieved were manually scanned to identify further articles missed by electronic searching. No language restrictions were applied provided there was an abstract available in English or Chinese. See Appendix A for search strategy.

### 2.2. Selection Criteria

Three reviewers (Lizhou Liu and Leon Mabire for English databases; Lizhou Liu and Jundong Wang for Chinese databases) independently screened for potential articles and resolved disagreements by discussion. Where necessary, full papers were obtained for final decision. “Systematic review” was defined methodologically as reviews with a systematic analysis, either meta-analysis or best-evidence synthesis.

Articles were included if they were systematic reviews of randomized controlled trials (RCTs) that evaluated the effectiveness of acupuncture compared to controls for any type of LBP (acute/subacute LBP: <3 months; chronic LBP: ≥3 months) [35], using at least one of the following outcome measures: pain relief, functional improvement, overall improvement, or effectiveness rate (frequently used in Chinese articles as the ratio of “cured and improved” to the total number of the group). Systematic reviews assessing needle acupuncture were considered irrespective of intervention styles. Acupuncture was described as a treatment procedure involving one or more needles penetrating into the skin without an injection [28]. The modes of acupuncture were not restricted: Traditional Chinese Medicine (TCM) acupuncture that adhered to energetic philosophical theory and Western medical acupuncture (e.g., dry needling) based on contemporary scientific principles were both eligible. However, articles relating to techniques similar to acupuncture but without needle insertion (e.g., laser acupuncture, acupressure, acupoint embedding, and auricular seeds) were excluded.

Systematic reviews were eligible that included control groups treated with sham acupuncture, no treatment/waiting list, conventional therapy, or sham therapy/placebo (e.g., sham laser or sham transcutaneous electrical nerve stimulation). Only systematic reviews in which the effectiveness of acupuncture could be isolated were included: that is, systematic reviews that tested effects of acupuncture alone over control comparators or evaluated acupuncture as an adjunct to other therapies and compared to the other therapies alone were included.

The following were excluded: (1) review comments, overviews of evidence, guidelines, editorials; (2) systematic reviews that included trials other than RCTs; (3) systematic reviews without formal analysis contained in the methods; (4) systematic reviews with no control comparisons, where only different forms of acupuncture were compared; (5) systematic reviews that only evaluated the cost-effectiveness of acupuncture; (6) systematic reviews that assessed the combined effects of acupuncture with other treatments over control interventions; and/or (7) systematic reviews where a series of conservative therapies or musculoskeletal conditions were investigated, but there was no separate data available for effectiveness of acupuncture for LBP.

### 2.3. Data Extraction

Data from articles written in English were extracted independently by two reviewers (Lizhou Liu and Leon Mabire) using a standardized spreadsheet designed to record descriptive characteristics, methodological quality of primary studies, acupuncture style and adequacy, data analysis approaches, main results, and conclusions. Articles written in Chinese were extracted independently by Lizhou Liu and Jundong Wang in the same manner. When retrieving articles published in other languages, translations of essential details were obtained for data extraction; differences during this process were settled by discussion with reference to the original papers.

The methodological qualities of the original (RCT) studies were then extracted, and quality levels were recorded as “Low” or “High,” based on the judgments made by the authors of the respective systematic reviews. As recommended, for systematic reviews using the Jadad scale [36], a score of two points or less (out of a maximum of five) represented poor quality; for systematic reviews using the PEDro scale [37], score of three points (out of a maximum of ten) was considered the cut-off for low quality [38, 39]; for systematic reviews which used criteria list from Method Guidelines for Systematic Reviews in the Cochrane Back Review Group [40–42], low quality was defined as a score less than 5/10, 6/11, and 6/12 for the versions of 1997, 2003, and 2009, respectively; two systematic reviews used additional requirements to judge the quality; the related information was extracted: one review [16] emphasized the necessity of adequate randomization and/or allocation concealment of RCTs to be of high quality and another review [25] weighted the importance of low drop-out at follow-up, between group statistical tests, and adequate power. When no clear judgment was available from the authors, we (Lizhou Liu and Leon Mabire) made determinations in accordance with the guidelines for each scale; the included RCTs were judged as “Low” quality if over 50% of these were of high risk.

The external validity of the included systematic reviews was assessed, using the Revised Standards for Reporting Interventions in Clinical Trials of Acupuncture (STRICTA) [43], a recommendation for reporting related characteristics of acupuncture trials. Furthermore, in order to identify whether the adequacy of acupuncture was considered in the analysis and the conclusions of the reviews, assessments concerning acupuncture adequacy in individual systematic reviews were extracted.

### 2.4. Quality Assessment

Quality assessment of systematic reviews was performed using the Assessment of Multiple Systematic Reviews (AMSTAR) criteria, a validated instrument with good construct validity and reliability [44]. It comprised 11 items, scored as “Yes,” “No,” “Cannot Answer,” or “Not Applicable” on a checklist. Before the assessment started, each topic of AMSTAR was intensively discussed to achieve homogeneity in the following procedure. Two reviewers (Lizhou Liu and Leon Mabire) independently rated the methodological quality, scored one point for item assigned “Yes,” and then calculated the overall score of each systematic review. The kappa statistic was used to measure the agreement level between the two reviewers; kappa index less than 0.4 reflected poor agreement, 0.4 to 0.75 fair agreement, and over 0.75 excellent agreement [45]. Again, consensus was reached by discussion between two reviewers and an independent decision was obtained from a third author (George David Baxter) if necessary. We considered the reviews as low quality if the total score was 4 or lower, moderate quality if the score was between 5 and 7, and high quality if the score was 8 or higher [46]. The classification of quality was used to grade the strength of the evidence in conclusions.

### 2.5. Data Synthesis

As the outcomes of the systematic reviews were likely to vary due to factors such as chronicity, the range of control groups, and the follow-up time points used,* a priori*, we assigned systematic reviews to predefined subgroups according to the following criteria:

1. *LBP chronicity*: acute/subacute LBP: <3 months, chronic LBP: ≥3 months;
2. *control comparisons*: sham acupuncture involving nonpenetration or superficial insertion of needles; sham therapy which was physiologically inert; no treatment or waiting list; conventional therapy including usual care or any active treatment other than acupuncture; and acupuncture added to conventional therapy compared to the conventional therapy alone;
3. *outcome measures*: primary outcome: pain relief and functional improvement, and secondary outcome: overall improvement or effectiveness rate;
4. *time points*: short-term follow-up: <3 months after randomization; intermediate follow-up: ≥3 months and <1 year; long-term follow-up: ≥1 year [41].

Outcomes of subgroup comparisons were summarized and appraised. A narrative synthesis of the evidence was presented to generate final conclusions, taking into account the methodological quality, the outcomes, and the total numbers of systematic reviews that reported consistent results (effectiveness or noneffectiveness). We assigned larger impact weights of outcomes for systematic reviews with higher quality and determined the overall conclusion according to the majority (>50%) of systematic reviews with consistent outcomes.

### 2.6. Sensitivity Analysis

A sensitivity analysis was conducted by excluding the systematic reviews of low quality and then repeating the aforementioned analysis.

### 2.7. Clinical Relevance

In order to identify whether any observed differences between acupuncture and control groups were clinically relevant, the pooled effect magnitudes of the meta-analyses were recorded.

For systematic reviews which used the same outcome instruments, an anchor-based approach using mean differences in pain intensity and functional disability was used to address clinical relevance. The minimal important changes (MIC) for pain relief were defined as 15/100 for the Visual Analogue Scale (VAS), 2/10 for the Numerical Rating Scale (NRS) [47]; for functional improvement these were defined as 5 points for the Roland Disability Questionnaire (RDQ), 10 points for the Oswestry Disability Index (ODI), and 20 points for the Quebec Back Pain Disability Questionnaire (QBDQ) [47].

For systematic reviews that used different scales, distribution-based methods were used to operationalize clinical relevance, that is, weighted mean difference (WMD) or standardized mean difference (SMD) for continuous outcomes, and odds ratio (OR) or relative ratio (RR) for dichotomous outcomes. Findings were determined to be clinically relevant based on the effect size only, and the degree was specified in accordance with the 2009 Updated Guidelines for Systematic Reviews in the Cochrane Back Review Group [42]: (1) small: WMD < 10% of the VAS scale; SMD < 0.5; RR < 1.25; (2) medium: WMD ≥ 10% and ≤20% of the VAS scale; SMD ≥ 0.5 and <0.8; RR ≥ 1.25 and ≤2.0; (3) large: WMD > 20% of the VAS scale; SMD ≥ 0.8; RR > 2.0.

## 3. Results

### 3.1. Systematic Review Selection

**Figure 1:**
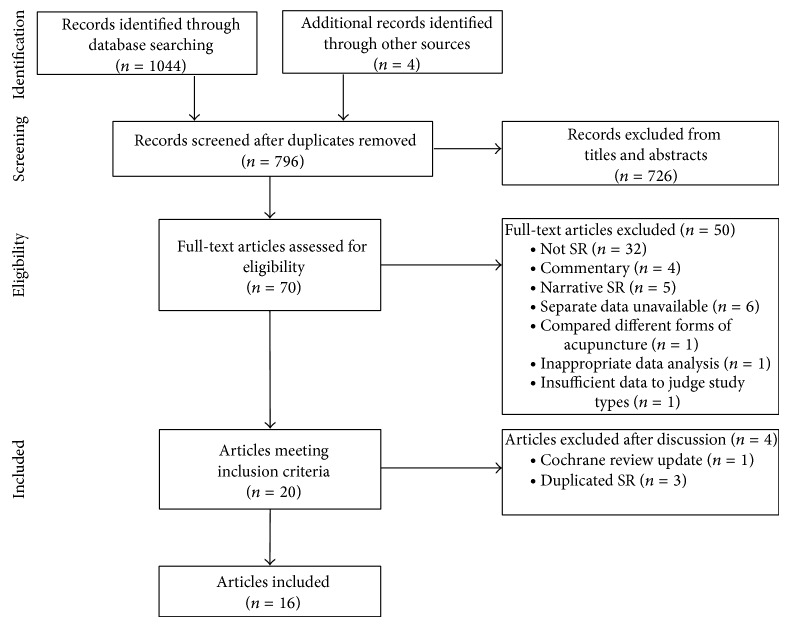
Flow diagram of literature search. SR: systematic review; RCTs: randomized controlled trials.

Our search strategy resulted in the identification of 1044 records; after excluding duplicates, 796 publications were manually screened by titles and abstracts, and 70 papers were eligible for inclusion. On the basis of review of full-text articles, 50 systematic reviews were excluded and 20 met our inclusion criteria. After discussion, four reviews were subsequently removed due to one for the Cochrane reviews update [48] and three for the same reviews reported as journal articles [29, 49, 50]. Thus a total of 16 systematic reviews were finally included in this overview (Figure 1).  Appendix B gives reasons for exclusion.

### 3.2. Systematic Review Characteristics

The main characteristics of systematic reviews are displayed in Table 1. Thirteen systematic reviews performed meta-analyses [16–23, 26–28, 30, 31], one conducted best-evidence syntheses [24], and two used both quantitative and qualitative analyses [25, 29]; one of which based conclusions on the qualitative analysis only [25]. The included systematic reviews were published between 1998 and 2013; twelve were published in English, three in Chinese, and one in Japanese. The number of RCTs included in the systematic reviews varied widely, ranging from 5 to 35 studies. Five systematic reviews were on chronic LBP [17, 19, 22–24], one on acute LBP [16], eight included a mixed group of participants with acute, subacute, and chronic LBP [18, 20, 25, 27–31], and the remaining two did not specify chronicity [21, 26]. In regard to the cause of LBP, seven systematic reviews only included RCTs of nonspecific LBP [16, 17, 22, 23, 25, 27, 29], three systematic reviews in Chinese exclusively focused on RCTs of lumbar intervertebral disc herniation (LIDH) [18, 21, 26], one involved RCTs of both specific and nonspecific LBP [20], and the remaining five did not clearly state the type of LBP [19, 24, 28, 30, 31].

### 3.3. Acupuncture Details

The majority of systematic reviews (*n* = 12/16) indicated the types of acupuncture assessed: six investigated TCM acupuncture as the exclusive intervention [17, 18, 21, 22, 26, 30] and six focused on both TCM and Western medical acupuncture [16, 24, 25, 28, 29, 31]. However, the reporting of acupuncture trials was of poor quality: only one systematic review clearly presented the extracted details of acupuncture in accordance with the STRICTA guidelines [16]. Assessment of the adequacy of acupuncture treatment was rarely considered in the systematic reviews: only four (25%) provided explicit criteria for judging whether the acupuncture intervention was adequate or not [16, 25, 29, 31].

### 3.4. Methodological Quality

The quality assessment scales of the original studies varied across the included systematic reviews; nine used the criteria list from the Method Guidelines for Systematic Reviews in the Cochrane Back Review Group [16, 19, 20, 22, 24, 25, 27–29], three adopted the modified Jadad scale [21, 28, 31], two selected the Cochrane risk of bias tool [17, 18], and the remaining two employed the PEDro scale and the assessment model used by Jüni et al. [51], respectively [23, 26]. Overall, the quality of RCTs was relatively low: of the 15 systematic reviews that provided quality assessment, nine were considered to include limited quality of RCTs by primary authors and by our two reviewers (Lizhou Liu and Leon Mabire) [16–18, 21, 22, 24, 25, 28, 29].

Agreement of the two reviewers for quality assessment of systematic reviews using AMSTAR was regarded as excellent (kappa index was 0.797) for independent reviews. After discussion the reviewers reached consensus giving a kappa index of 1.  Table 2 provides an overview of the assessment results. The overall scores ranged from 2 to 9 (out of a maximum of 11); three systematic reviews were considered as high quality [28, 29, 31], eight as moderate quality [16–22, 26], and five as low quality [23–25, 27, 30]. The number of reviews satisfying the criteria for individual items varied widely: four items were satisfied by over 75% of the systematic reviews, namely, Item 2, the duplicate processes of study selection and data extraction (*n* = 13); Item 3, the comprehensive literature search strategy (*n* = 12); Item 7, the scientific quality assessment of the included studies (*n* = 15); and Item 9, the appropriate methods of meta-analysis (*n* = 13). In contrast, three items accounted for the major methodological limitations: Item 11, the interest conflict statement, was not met by any of the systematic reviews but one, which indicated source of funding for the review as well as for the included studies; Item 1,* a priori* design requirement, and Item 5, presenting a list of excluded studies in addition to included studies, were rarely reported in two systematic reviews.

### 3.5. Outcomes

Because of the inconsistent definition of follow-up time points in individual systematic reviews, only short-term (<3 months) comparisons could be assessed. The duration of “short-term” was in the range from 6 weeks [27, 28] to 3 months [20, 24, 25, 29]. Subgroup analyses were conducted as planned, except for the comparison between acupuncture and conventional therapy due to high heterogeneity: while some systematic reviews mixed various conventional treatments as one control arm, some considered different interventions as independent control groups; thus pooling the data for conventional therapy was impossible. For secondary outcomes, data were sparse and insufficient for drawing conclusions. Ultimately, comparisons of four control groups for pain and functional outcomes at short-term follow-up were made. Tables 3 and 4 present the pooled effects for the related outcomes.

#### 3.5.1. Acute/Subacute LBP

There were two systematic reviews with meta-analyses, which provided sufficient data for comparison [16, 20]. 

*Acupuncture versus Sham Acupuncture*. In the two systematic reviews, sham acupuncture meant mimicked nonpenetration on the same acupoints used for genuine acupuncture.

*Pain Relief*. Two systematic reviews produced conflicting conclusions. Furlan and colleagues performed one meta-analysis (moderate quality) with two RCTs (one for acute LBP and one for subacute LBP) which indicated that the effectiveness of acupuncture did not differ from sham acupuncture for posttreatment pain intensity [20]. In contrast, another systematic review of moderate quality based on two RCTs of low risk of bias (one was common to both systematic reviews) found that there was a statistically, but not clinically relevant, difference immediately after intervention between a single session of acupuncture and sham acupuncture for individuals with acute LBP (MD = −9.38, 95% CI: −17.00 to −1.76; *P* = 0.02; *I* ^2^ = 27%) [16]. 

*Functional Improvement*. Two systematic reviews of moderate quality both yielded a negative result that suggested similar benefits from real and sham acupuncture needling. Furlan et al. reported that acupuncture and the sham acupuncture were not significantly different at 3-month follow-up [20]. Similarly, Lee et al. using three studies of low risk of bias found no significant difference between real and sham acupuncture for either acute LBP (2 studies, 100 participants) or subacute LBP (1 study, 48 participants) [16].

#### 3.5.2. Chronic LBP

*Acupuncture versus Sham Acupuncture*. There was wide variation in the definition of sham acupuncture, including (1) mimicked nonpenetration on identical acupoints used for verum procedure [19, 28]; (2) superficial insertion outside acupoints without stimulation [17, 19, 25, 28]; (3) 2% lidocaine injection at nonacupoints plus superficial insertion without stimulation [25].

*Pain Relief*. Four systematic reviews reported contradictory outcomes [17, 19, 25, 28], but those that supported real acupuncture were of higher quality overall, with more careful consideration of pooling of data. Two systematic reviews (one of high quality and one of moderate quality) found statistically significant effects of authentic acupuncture in relieving pain compared with sham [17, 28], while another two (one of moderate quality and one of low quality) showed equivalent outcomes [19, 25]. Of the two systematic reviews that found data in favor of acupuncture, a moderate clinically relevant effect size was observed at the end of treatment [28]. Another systematic review [52] used individual patient data meta-analysis and also demonstrated that genuine acupuncture was more efficacious than sham acupuncture, with an effect size of standardized differences being 0.20 (95% CI: 0.09 to 0.31) in the sensitivity analysis; however, this review was excluded from our overview because its primary analysis investigated the effectiveness of acupuncture in treatment of neck pain and back pain as a whole.

*Functional Improvement*. The four eligible systematic reviews consistently found no evidence supporting the effectiveness of acupuncture over sham acupuncture [17, 19, 25, 28].

*Acupuncture versus Sham Therapy*. Sham therapy groups included either a combination of sham acupuncture and sham TENS [22, 24, 29] or a mix of sham interventions [20].

*Pain Relief*. While five systematic reviews reported contradictory results [20, 22–24, 29], the three of higher quality suggested that individuals who received acupuncture experienced lower levels of pain than their counterparts who received sham treatments. One systematic review with best-evidence synthesis (low quality) failed to demonstrate the positive benefits of acupuncture over sham interventions [24]; in contrast, three of the four systematic reviews with meta-analysis (one of high quality and two of moderate quality) revealed that acupuncture compared to sham therapies led to significantly lower pain intensity at short-term follow-up [20, 22, 29]. Effect sizes were small to moderate; WMD ranged from −5.88 (95% CI, −11.20 to −0.55) at 1 month [22] to −17.79 (95% CI, −25.50 to −10.07) at 3 months [29]. 

*Functional Improvement*. Relatively clear consensus emerged among the four systematic reviews that acupuncture did not significantly differ from sham therapy in reducing disability [20, 22, 24, 29]. While the evidence of three systematic reviews (one of high quality, one of moderate quality, and one of low quality) seemed to be negative [20, 24, 29], one systematic review of moderate quality using meta-analysis demonstrated that subjects receiving acupuncture had significantly fewer functional limitations, but the effect size was small [22]. 

*Acupuncture versus No Treatment*. There was little agreement in the description of “no treatment” among the included systematic reviews. While seven systematic reviews defined no treatment as waiting list control (i.e., no care while waiting for acupuncture) [17, 20, 22, 24, 25, 28, 29], one systematic review included waiting list control as well as another form of treatment comparison, which assessed the effects of adding acupuncture to other therapies, compared with other therapies alone [19]. As there was a significant difference between the two categories, the latter was not included for analysis. 

*Pain Relief and Functional Improvement*. All seven systematic reviews (two of high quality, three of moderate quality, and two of low quality) indicated that acupuncture was superior both in reducing pain and improving function for chronic LBP [17, 20, 22, 24, 25, 28, 29]. Moreover, the overall effect sizes were medium to large for both outcome measures. 

*Acupuncture in addition to Conventional Therapy versus Conventional Therapy Alone*. Conventional therapy consisted of usual care [17] or other treatments such as physiotherapy, medications, or exercises [22, 24, 25, 29].

*Pain Relief and Functional Improvement*. All five systematic reviews (one of high quality, two of moderate quality, and two of low quality) consistently supported acupuncture as an adjunct to conventional care in the treatment of LBP [17, 22, 24, 25, 29]. For measures of pain, two of the three systematic reviews that provided pooled effects demonstrated the differences in effect were medium to large [22, 29], and the remaining one reported statistically but not clinically significant (15/100 for VAS as MIC for pain) effects (MD = −13.99, 95% CI: −20.48 to −7.50; *P* < 0.000; *I* ^2^ = 34%) [17]. For measures of function, three systematic reviews showed large effect sizes [17, 22, 29].

### 3.6. Sensitivity Analysis

The results of sensitivity analysis are given in Table 5. As planned, after excluding five systematic reviews of low quality, eleven systematic reviews were subsequently included for analysis [16–22, 26, 28, 29, 31]. The current conclusions regarding the effectiveness of acupuncture compared with no treatment and acupuncture in addition to other conventional therapies for chronic LBP did not change with the exclusion of two systematic reviews [24, 25]. Conclusions regarding the effectiveness of acupuncture compared with sham therapy for pain intensity with the two reviews excluded would be consistently positive, in that acupuncture has a more favorable effect. Furthermore, the conclusions regarding the effectiveness of acupuncture compared with sham acupuncture would point to stronger evidence that real acupuncture is more efficacious than sham for self-reported pain, as the majority of systematic reviews were in favor of true acupuncture.

## 4. Discussion

### 4.1. Statement of Main Findings

The purpose of the present overview was to critically evaluate the evidence from systematic reviews and to provide a rigorous and objective summary from the best credible evidence concerning the effectiveness of acupuncture in the treatment of LBP. Overall the analysis suggests that, (1) for acute LBP, there exists inconsistent evidence that acupuncture has a more favorable effect than sham acupuncture in relieving pain but consistent evidence that acupuncture does not significantly differ from sham acupuncture in improving function; (2) for chronic LBP, consistent evidence found that acupuncture provides short-term clinically relevant benefits on pain relief and functional improvement when compared with no treatment or when acupuncture is added to another conventional intervention; (3) for chronic LBP, it seems that genuine acupuncture produces a clinically significant reduction in pain when compared to sham acupuncture and sham therapy at short-term follow-up, but no impact on functional limitation.

### 4.2. Internal Validity of the Included Systematic Reviews

The methodological quality assessment of the included systematic reviews reveals there are common areas for improvement. Of the sixteen systematic reviews assessed, only three met the preset “high quality” level (≥8/11 on the AMSTAR checklist). Given that systematic reviews are not equally reliable due to variable quality and the ones with insufficient quality are likely to have biased findings [53], it seemed reasonable to place a greater weighting on systematic reviews of higher quality in drawing conclusions. Furthermore, in order to address the impact of low quality systematic reviews on the overall conclusions, a sensitivity analysis was conducted by excluding all the systematic reviews of low quality (≤4/11 on the AMSTAR checklist); under these conditions, conclusions concerning the effectiveness of acupuncture for LBP pointed to stronger evidence showing that acupuncture is an effective treatment for patients with LBP.

Because AMSTAR provides for qualitative rather than quantitative assessments, there is no consensus on the definition of quality levels. One of the challenges with using AMSTAR in this way is that the interpretation depends on the total score, and there is no weighting of individual items. However the cutoff point we selected was the one employed in the overviews of reviews conducted by the International Collaboration on Neck (ICON) working group that evaluated evidence for the management for neck pain [46, 54] and is consistent with that from the National Institute for Health Research (NIHR): the systematic review of the highest quality [29] in our rating system was considered to have a very low risk of bias according to the NICE guidelines [14]. For other studies use of AMSTAR produced a low quality rating, for example, Yuan et al. [25], whereas in a separate review of systematic reviews [55] this review was rated as good (the higher possible rating) using a different rating scale; some of these differences can be explained by AMSTAR including “new” items considered potential sources of bias (language and publication bias, not included in older scales) or differences between raters in how questions on both scales are interpreted. We did not attempt to contact authors of each systematic review to determine whether certain methodological items were completed (and perhaps not reported), and given the relative newness of the AMSTAR scale and the journal space limitations, this could have altered the scores for some of the systematic reviews.

### 4.3. External Validity of the Included Systematic Reviews

Overall, the external validity of the included systematic reviews was limited: only one systematic review reported the characteristics of interventions on the basis of STRICTA, while the remaining showed considerable heterogeneity in terms of data presentation. This may in part be due to the lack of endorsement of the guidelines: currently few journals have endorsed the STRICTA guidelines and even fewer have made these mandatory requirements for publication [56] or could simply be explained by space limitations for systematic reviews which may already be quite lengthy.

As an official extension of the Consolidated Standards for Reporting Trials (CONSORT) [43], STRICTA is mainly designed for clinical trials to improve the completeness of intervention reporting; however it is also useful for authors of systematic reviews [57]. From our experience, systematic reviews that include complete detail on the STRICTA items could furnish researchers with a reasonable and transparent interpretation for clinical heterogeneity and provide health professionals with greater confidence when using the related evidence in their routine practice. Furthermore, it may also facilitate the development of criteria for assessing the adequacy of acupuncture where there is little agreement [58]; indeed, this might be the very reason for the scarce description of acupuncture adequacy assessment in the included systematic reviews as only 4/16 mentioned this related information. We strongly encourage future systematic reviews and trial studies to apply STRICTA and adhere to the statement and hence enhance the scientific quality of acupuncture research.

### 4.4. Problems with Sham Acupuncture

Given that systematic reviews as well as RCTs typically set sham acupuncture as a control arm to investigate the specific effects of acupuncture, it is worth exploring the validity of this approach. In our overview, sham acupuncture mainly included “sham acupuncture” which used needles of blunt tips without skin penetration, or “minimal acupuncture” which inserted needles outside acupoints and/or superficially. This might be an appropriate control according to the Chinese meridian theory since neither noninsertion nor insertion at nonacupoints would elicit any therapeutic effects; however other forms of Asian medicine use noninsertion needling for therapeutic effects, for example, in Japan. In addition, accumulating evidence from recent research argues that neither of the two forms are fully inert from a physiological perspective, because skin touching as described would induce emotional and hormonal reaction [59], activate afferent nerve fibers, and cause deactivation of limbic structures resulting in part at least, if not all, of the specific effects of the needles [60, 61]. Therefore, the validity of current techniques of sham acupuncture remains unclear, and results obtained from such comparison should be interpreted with caution [61].

Although improved function was observed in both verum and sham acupuncture groups, none of the six systematic reviews found statistically significant intergroup differences [16, 17, 19, 20, 25, 28]. Beyond the issue of limited differences between genuine and sham acupuncture (as outlined above), the two forms of acupuncture may be associated with potent nonspecific effects that could lead to equivalent functional improvements, that is, psychological (placebo) effects as patients' expectancy, and patient-clinician relationship [52, 62]. Hence, it raises the question about whether it is necessary to separate the specific from nonspecific effects of acupuncture.

Based on the recent evidence, the answer might be “no” [60, 63]. Assuming that clinical improvement can be achieved independently of affective factors challenges the emphasis of TCM on the holistic integration of “body and mind”; equally, Western medicine has accepted a “biological-psychological-social” model as a frame of reference for low back pain [60, 63]. Furthermore, diagnosis of nonspecific LBP is mainly based on patients' subjective description of symptoms, rather than specific clinical diagnostic tests [27], and prognosis is predominantly associated with affective components related to patients' beliefs and expectations that the treatment will be effective [59]. Sham acupuncture attempts to isolate the physiological effects and is therefore not a perfect research choice. Moreover, in routine clinical practice, physicians and patients do not make decisions between true and sham acupuncture; instead they pay more attention to the choice between treatments, acupuncture, or other therapies [52]. Thus it could be argued that future grant-aided research will be more cost-effective if the research focus shifts from disentangling the effects of true and sham acupuncture, to exploring the effectiveness of acupuncture over other conventional therapies which have been proved to be effective for LBP (i.e., best current treatment).

### 4.5. Strengths of This Overview

We have made efforts to minimize the risk of bias in every step of this overview. Firstly, for literature identification, we used systematic, comprehensive, and independent search strategies over a wide range of English and Chinese electronic databases, without restriction of language and year of publication. Secondly, for quality assessment, we engaged independent reviewers (Lizhou Liu and Leon Mabire) from diverse academic backgrounds who have participated in Cochrane training for systematic reviews to use the AMSTAR checklist with added quantitative rating criteria, and agreement between reviewers on validity assessment was excellent. Thirdly, for data synthesis, we performed subgroup analysis stratified by LBP chronicity, control comparisons, and outcome measures to address the influence of heterogeneity. Fourthly, for conclusion generation, we synthesized results from systematic reviews with formal analysis methodology to guarantee reliability of the conclusions and performed sensitivity analysis to test scientific robustness. Finally, for evidence applicability, we considered outcomes of clinical (as well as statistical) relevance to increase clinical applicability.

### 4.6. Implications for Practice and Research

For acute LBP, we could not make firm conclusions about the effectiveness of acupuncture on the basis of only two systematic reviews, and thus there is a need for future research to make more definitive recommendations. For chronic LBP, consistent evidence shows that acupuncture is more effective for pain relief and functional improvement at short-term follow-ups when compared to no treatment or when used with other conventional therapies; these results had medium to large clinical effects. According to these findings, it is encouraging to note that acupuncture, either used in isolation or as an adjunct to other interventions, has been demonstrated as an effective clinical option for patients with chronic LBP and should be advocated in routine clinical practice. Considering the intractable nature of LBP, more effective, comprehensive treatment options, which might include acupuncture, are needed to optimize current management [19].

In the context of future research, there is a need for higher quality RCTs and systematic reviews which strictly adhere to relevant guidelines and particularly STRICTA guidelines to improve both internal and external validity. Furthermore, as empirical evidence has indicated that the response of acupuncture is associated with an adequate dose of stimulation [64], as for pharmaceutical therapy [31, 65], future systematic reviews should assess the adequacy of acupuncture treatment and consider the results in their conclusions as identified in some of the included reviews [16, 25, 29, 31]. Future systematic reviews should also grade the strength of evidence by adopting accepted instruments such as the Grading of Recommendations Assessment, Development and Evaluation (GRADE) [66] to enable rigorous recommendations to evidence-users. From the available outcomes of the included systematic reviews, some subgroups could not be analyzed; therefore, more research is needed to focus on areas where there is little evidence, for example, acupuncture for acute LBP, acupuncture for other outcome measures besides pain and function, acupuncture in long-term follow-up, and acupuncture compared to conventional therapy. Future research should also investigate the essential characteristics of acupuncture for its effectiveness (i.e., mode of administration, pattern of stimulation, choice of needles, number of sessions, duration of treatments, use of cointerventions, and experience of practitioners) and determine the potential relevance of such characteristics to the effectiveness of acupuncture for LBP.

## 5. Conclusions

Based on seven systematic reviews (two of high quality, three of moderate quality, and two of low quality), acupuncture is more clinically effective in pain relief and functional improvement than no treatment at short-term follow-up. Based on five systematic reviews (one of high quality, two of moderate quality, and two of low quality), acupuncture as an adjunct to conventional therapy provides short-term clinically relevant improvements in pain and functional measures for the treatment of chronic low back pain. More efforts are needed to improve both internal and external validity of systematic reviews and RCTs in this area.

## Figures and Tables

**Table 1:** Characteristics of systematic reviews.

Authors (date)/ country	Condition	Number of RCTs (patients) included	Quality of original studies scale/level	Acu style of SR	Acu adequacy assessment of original studies	Quality of SR	Data analysis methods	Comparisons	Reported results		Authors conclusions/comments [notes]
									Pain relief	Functional improvement	
Lee et al. (2013) [16]/Korea	Acute NSLBP	11 (1139)	2009 CBRGC and additional criteria (5/11) Low	TCM, Western	Judged by two experts about two questions	6	Meta- analysis	(1) Sham acu (2) Medications	+ #	= NR	Acupuncture may be more effective than medication for symptom improvement or relieve pain better than sham acupuncture in acute NSLBP/include Asian database. Strict criteria of being high quality studies. Emphasize the impact of language restriction and Chinese studies [overall improvement (+) compared to medications]

Lam et al. (2013) [17]/Ireland	Chronic NSLBP	32 (5931)	Cochrane risk of bias tool (7/32) Low	TCM	NR	6	Meta- analysis	(1) Sham acu (2) No treatment (3) Plus usual care (4) Medications (5) TENS	+ + + + =	= + + + NR	Acupuncture may have a favorable effect on pain and function on chronic NSLBP/high heterogeneity of included studies. Confused subgroups. Clear definition of clinical significance.

Wu et al. (2013) [18]/China	LIDH	6 (540)	Cochrane risk of bias tool (NR) Low	TCM	NR	5	Meta- analysis	Various controls (oral medications, traction, medication injection, or transfusion)	=	NR	Acupuncture is effective and safe for LIDH/article in Chinese. Restricted publication status. No subgroup comparison provided. Quality of evidence evaluated by GRADE [noneffectiveness rate (−)]

Xu et al. (2013) [19]/China	Chronic LBP	13 (2678)	2003 CBRGC (12/13) High^⊚^	NR	NR	5	Meta- analysis	(1) No treatment (2) Conventional therapy (3) Sham acu	+ + =	+ + =	Acupuncture is an effective treatment for chronic LBP but may due to nonspecific effects/exclude Asian database other than Chinese. No key studies characteristics provided [no treatment group included blank treatment comparison]

Furlan et al. (2012) [20]/ Canada	LBP	33 (NR)	2009 CBRGC (NR) NR	NR	NR	5	Meta- analysis	*For acute/subacute NSLBP* (1) Sham acu *For chronic NSLBP* (1) Sham therapy (2) No treatment (3) Medications (4) Manipulation (5) Usual care	= + + = − +	= = + = NR +	Same as reported results/not specific to acupuncture. No studies characteristics provided. Quality of evidence evaluated by GRADE.

Li et al. (2010) [21]/China	LIDH	5 (718)	Jadad (2/5) Low	TCM (electro)	NR	5	Meta- analysis	Various controls (oral medications or physiotherapy)	+	NR	Electroacupuncture is effective and safe for LIDH/article in Chinese. Restricted publication status. No subgroup comparison provided. Poor quality of meta-analysis (repeatedly pooled the same study in one analysis) [effective rate (+)]

Rubinstein et al. (2010) [22]/Netherlands	Chronic NSLBP	20 (5590)	2003 CBRGC (8/20) Low	TCM	NR	5	Meta- analysis	(1) Sham therapy (2) No treatment (3) Plus conventional therapy (4) Usual acre	+ + + +	+ + + +	Acupuncture provides short-term clinically relevant effects when compared with no treatment or added to other therapies/not specific to acupuncture. No studies characteristics provided. No heterogeneity present. Quality of evidence evaluated by GRADE.

Machado et al. (2009) [23]/Australia	NSLBP	4 (149)	PEDro (4/4) High^⊚^	NR	NR	3	Meta- analysis	Placebo controls	=	NR	Acupuncture is not more effective than placebo/not specific to acupuncture. No studies characteristics provided. No heterogeneity present.

Ammendolia et al. (2008) [24]/ Canada	Chronic LBP	19 (5001)	2003 CBRGC (10/19) Low	TCM, Western	NR	4	Best-evidence synthesis	(1) Waiting list (2) Plus conventional therapy (3) Conventional therapy (4) Sham therapy	+ + = #	+ + = =	Same as reported results/lack rigorous format of SR.

Yuan et al. (2008) [25]/UK	NSLBP	23 (6359)	2003 CBRGC and additional criteria (6/23) Low^⊚^	TCM, Western	Compared to textbooks, surveys, and primary reviews	4	Meta- analysis, best-evidence synthesis	(1) No treatment (2) Plus Conventional therapy (3) Sham acu (4) Conventional therapy	+ + = #	+ + = #	Acupuncture versus no treatment and as an adjunct to conventional care should be advocated in the European Guidelines for chronic LBP/exclude non-English articles. No heterogeneity present. No overall effect size of pooled studies provided. Strict criteria of being high quality studies. Clear definition of clinical significance.

Li et al. (2008) [26]/China	LIDH	5 (547)	Jüni (NR) High	TCM (electro)	NR	7	Meta- analysis	Various controls (oral medications or physiotherapy)	+	+	Electroacupuncture is effective and safe for LIDH/article in Chinese. No subgroup comparison provided.

Keller et al. (2007) [27]/Norway	NSLBP	7 (528)	2003 CBRGC and Jadad (NR) High	NR	NR	2	Meta- analysis	Various controls (sham therapy or no treatment)	+	NR	Acupuncture has modest effect for chronic LBP/not specific to acupuncture. No subgroup comparison provided.

Manheimer et al. (2005) [28]/USA	LBP	33 (2300)	1997 CBRGC and Jadad (12/33, 17/33) Low	TCM, Western	NR	8	Meta- analysis	*For acute LBP* *For chronic LBP* (1) Sham acu (2) No treatment (3) Conventional therapy (4) Manipulation	⊥ + + ⊥ −	⊥ = + ⊥ −	Acupuncture effectively relieves chronic low back pain. No evidence suggests acupuncture is more effective than other active therapies/exclude Chinese database. Restricted publication status. Clear definition of clinical significance.

Furlan et al. (2005) [29]/Canada	NSLBP	35 (2861)	2003 CBRGC (14/35) Low	TCM, Western	Judged by three experienced acupuncturists based on four questions	9	Meta- analysis, best-evidence synthesis	*For acute NSLBP* *For chronic NSLBP* (1) Sham therapy (2) No treatment (3) Plus conventional therapy (4) Conventional therapy	⊥ + + + =	⊥ = + + =	No firm conclusions for acute LBP. For chronic LBP, acupuncture is more effective than no treatment or sham treatment. Acupuncture is not more effective than other conventional treatments. Acupuncture and dry needling may be useful adjuncts to other therapies/include Asian database. High heterogeneity of included studies. Subjective assessment of clinical relevance.

Zhu et al. (2002) [30]/China	LBP	9 (426)	NR	TCM	NR	2	Meta- analysis	Various controls (sham acu, TENS, medications, or physiotherapy)	+	NR	Acupuncture might be effective for LBP/article in Japanese. Include Japanese database. No subgroup comparison provided.

Ernst and White (1998) [31]/UK	Back Pain	12 (472)	Jadad (10/12) High	TCM, Western	Judged by six experienced acupuncturists	8	Meta- analysis	Various controls (sham acu, no treatment, TENS et al.)	NR	NR	Acupuncture is superior to various control interventions, but insufficient evidence to judge whether it is superior to placebo/no subgroup comparison provided [symptoms improve (+)]

LBP: low back pain; NSLBP: nonspecific low back pain; LIDH: lumbar intervertebral disc herniation; CBRGC: Cochrane Back Review Group Criteria; TCM: traditional Chinese medicine; acu: acupuncture; TENS: transcutaneous electrical nerve stimulation; NR: not reported.

⊚: judged by reviewers; #: conflicting evidence; ⊥: insufficient evidence.

+: more effective than; =: no difference found or not more effective than; −: less effective than.

**Table 2:** Methodological quality assessment of systematic reviews.

Authors (date)	1	2	3	4	5	6	7	8	9	10	11	Total
Lee et al. (2013) [16]	N	N	Y	Y	N	Y	Y	CA	Y	Y	N	6
Lam et al. (2013) [17]	N	Y	Y	N	N	Y	Y	Y	Y	N	N	6
Wu et al. (2013) [18]	N	Y	N	N	N	Y	Y	Y	Y	N	N	5
Xu et al. (2013) [19]	N	Y	Y	N	N	N	Y	N	Y	Y	N	5
Furlan et al. (2012) [20]	N	Y	Y	N	N	N	Y	N	Y	Y	N	5
Li et al. (2010) [21]	N	Y	Y	N	N	N	Y	Y	Y	N	N	5
Rubinstein et al. (2010) [22]	N	Y	N	N	N	N	Y	Y	Y	Y	N	5
Machado et al. (2009) [23]	N	Y	Y	N	N	N	Y	N	N	N	N	3
Ammendolia et al. (2008) [24]	Y	N	Y	N	N	Y	Y	N	NA	NA	N	4
Yuan et al. (2008) [25]	N	Y	Y	N	N	Y	Y	N	N	N	N	4
Li et al. (2008) [26]	N	Y	Y	Y	N	N	Y	Y	Y	Y	N	7
Keller et al. (2007) [27]	N	CA	N	N	N	N	Y	N	Y	N	N	2
Manheimer et al. (2005) [28]	N	Y	Y	Y	N	Y	Y	Y	Y	Y	N	8
Furlan et al. (2005) [29]	Y	Y	Y	N	Y	Y	Y	Y	Y	N	Y	9
Zhu et al. (2002) [30]	N	Y	N	N	N	N	N	NA	Y	N	N	2
Ernst and White (1998) [31]	N	Y	Y	Y	Y	Y	Y	N	Y	Y	N	8

Score	2	13	12	4	2	8	15	7	13	7	1	Mean = 5.25

(1) Was an “*a priori*” design provided? (2) Was there duplicate study selection and data extraction? (3) Was a comprehensive literature search performed? (4) Was the status of publication (i.e., grey literature) used as an inclusion criterion? (5) Was a list of studies (included and excluded) provided? (6) Were the characteristics of the included studies provided? (7) Was the scientific quality of the included studies assessed and documented? (8) Was the scientific quality of the included studies used appropriately in formulating conclusions? (9) Were the methods used to combine the findings of studies appropriate? (10) Was the likelihood of publication bias assessed? (11) Was the conflict of interests stated?

Y: yes; N: no; CA: cannot answer; NA: not applicable.

**Table 3:** Summary of positive results with meta-analysis, pain relief.

Comparator	Authors (date)	Number of RCTs (patients) pooled	Outcome measured time point	Effect estimate (MD, SMD, WMD) 95% confidence interval
Sham acupuncture	Lam et al. (2013) [17]	4 (287)	Immediately	(VAS) MD = −16.76 [95% CI, −33.33 to −0.19]
	Manheimer et al. (2005) [28]	4 (343)	<6 weeks	SMD = −0.58 [95% CI, −0.80 to −0.36]

Sham therapy	Furlan et al. (2012) [20]	10 (1727)	Immediately	WMD = −0.59 [95% CI, −0.93 to −0.25] (VAS: 1–10)
	Rubinstein et al. (2010) [22]	4 (918) 4 (1076)	<1 month <3 months	WMD = −5.88 [95% CI, −11.20 to −0.55] WMD = −7.27 [95% CI, −12.66 to −1.89]
	Furlan et al. (2005) [29]	4 (314) 2 (138)	Immediately <3 months	WMD = −10.21 [95% CI, −14.99 to −5.44] WMD = −17.79 [95% CI, −25.50 to −10.07]

No treatment	Lam et al. (2013) [17]	4 (2911)	Immediately	SMD = −0.72 [95% CI, −0.94 to −0.49]
	Xu et al. (2013) [19]	5 (NR)	>1 month	SMD = −0.64 [95% CI, −1.13 to −0.14]
	Furlan et al. (2012) [20]	3 (2684)	<3 months	WMD = −1.19 [95% CI, −2.17 to −0.21] (VAS: 1–10)
	Rubinstein et al. (2010) [22]	1 (214)	<3 months	WMD = −24.10 [95% CI, −31.52 to −16.68]
	Manheimer et al. (2005) [28]	8 (586)	<6 weeks	SMD = −0.69 [95% CI, −0.98 to −0.40]
	Furlan et al. (2005) [29]	2 (90)	<3 months	SMD = −0.73 [95% CI, −1.19 to −0.28]

Plus conventional therapy	Lam et al. (2013) [17]	4 (269)	Immediately	(VAS) MD = −13.99 [95% CI, −20.48 to −7.50]
	Rubinstein et al. (2010) [22]	2 (99) 3 (185)	<1 month <3 months	WMD = −9.80 [95% CI, −14.93 to −4.67] WMD = −16.91 [95% CI, −25.18 to −8.64]
	Furlan et al. (2005) [29]	4 (289) 3 (182)	Immediately <3 months	SMD = −0.76 [95% CI, −1.02 to −0.50] SMD = −1.10 [95% CI, −1.62 to −0.58]

VAS: Visual Analogue Scale; MD: mean difference; WMD: weighted mean difference; SMD: standardized mean difference; CI: confidence interval; NR: not reported.

**Table 4:** Summary of positive results with meta-analysis, functional improvement.

Comparator	Authors (date)	Number of RCTs (patients) pooled	Outcome measured time point	Effect estimate (MD, SMD) 95% confidence interval
Sham therapy	Rubinstein et al. (2010) [22]	1 (745) 3 (1044)	<1 month <3 months	SMD = −0.18 [95% CI, −0.32 to −0.04] SMD = −0.28 [95% CI, −0.41 to−0.16]

No treatment	Lam et al. (2013) [17]	3 (451)	Immediately	SMD = −0.94 [95% CI, −1.41 to −0.47]
	Xu et al. (2013) [19]	4 (NR)	>1 month	SMD = −0.58 [95% CI, −0.82 to −0.34]
	Furlan et al. (2012) [20]	1 (NR)	Immediately	MD = −8.20 [95% CI, −12.0 to −4.40]
	Rubinstein et al. (2010) [22]	1 (214)	<3 months	SMD = −0.61 [95% CI, −0.90 to −0.33]
	Manheimer et al. (2005) [28]	6 (NR)	<6 weeks	SMD = −0.62 [95% CI, −0.95 to −0.30]
	Furlan et al. (2005) [29]	2 (90)	<3 months	SMD = −0.63 [95% CI, −1.08 to −0.19]

Plus conventional therapy	Lam et al. (2013) [17]	3 (144)	Immediately	SMD = −0.87 [95% CI, −1.61 to −0.14]
	Rubinstein et al. (2010) [22]	2 (99) 4 (2824)	<1 month <3 months	SMD = −1.04 [95% CI, −1.46 to −0.61] SMD = −0.66 [95% CI, −0.74 to −0.58]
	Furlan et al. (2005) [29]	3 (173) 3 (173)	Immediately <3 months	SMD = −0.95 [95% CI, −1.27 to −0.63] SMD = −0.95 [95% CI, −1.37 to −0.54]

MD: mean difference; SMD: standardized mean difference; CI: confidence interval, NR: not reported.

**Table 5:** Sensitivity analysis.

Condition	Comparisons	Systematic reviews *N* =		Systematic reviews in favor of acupuncture *N* =	
Pain relief	Functional improvement	Pain relief	Functional improvement

Acute LBP	Sham acupuncture	2	2

Chronic LBP	(1) Sham acupuncture	3	3
	(2) Sham therapy	3	3
	(3) No treatment	5	5
	(4) Plus conventional therapy	3	3

Sensitivity analysis was performed by excluding systematic reviews of low quality in analysis.

LBP: low back pain.

**Table 6:** Excluded systematic reviews.

References	Reason for exclusion
White and Foell,* Evidence-Based Medicine*, vol. 18, no. 6, p. e56, 2013.	Not systematic review
Pennick and Liddle,* Cochrane Database of Systematic Reviews*, vol. 8, Article ID CD001139, 2013.	No separate data of acupuncture for back pain
Yu et al.,* Chinese Journal of Information on TCM*, vol. 19, no. 5, pp. 27–29, 2012.	No separate data available
Vickers et al., *Archives of Internal Medicine*, vol. 172, no. 19, pp. 1444–1453, 2012.	Unavailable separate data for back pain
Tan, Chengdu University of Traditional Chinese Medicine, Chengdu, China, 2012.	Inappropriate data analysis
Richards et al., *Acta Obstetricia et Gynecologica Scandinavica*, vol. 91, no. 9, pp. 1038–1045, 2012.	Narrative systematic review
Marlowe, *Primary Care: Clinics in Office Practice*, vol. 39, no. 3, pp. 533–546, 2012.	Not systematic review
Hutchinson et al., *Journal of Orthopaedic Surgery and Research*, vol. 7, p. 36, 2012.	Narrative systematic review
Ernst, *Focus on Alternative and Complementary Therapies*, vol. 17, no. 4, pp. 223-224, 2012.	Commentary
Amezaga Urruela and Suarez-Almazor, *Current Rheumatology Reports*, vol. 14, no. 6, pp. 589–597, 2012.	Not systematic review
Li, Shanghai University of Traditional Chinese Medicine, Shanghai, China, 2011.	Compared different forms of acupuncture
Katonis et al., *Hippokratia*, vol. 15, no. 3, pp. 205–210, 2011.	Not systematic review
Grazio and Balen*, Acta Clinica Croatica*, vol. 50, no. 4, pp. 513–530, 2011.	Not systematic review
Trigkilidas, *Annals of the Royal College of Surgeons of England*, vol. 92, no. 7, pp. 595–598, 2010.	Narrative systematic review
Slattengren,* Evidence-Based Practice*, vol. 13, no. 3, p. 13, 2010.	Not systematic review
Scott, *Journal of the Acupuncture Association of Chartered Physiotherapists*, no. 3, pp. 29–33, 2010.	Not systematic review
Furlan et al., *Evidence Report/Technology Assessment*, no. 194, pp. 1–764, 2010.	Duplicated systematic reviews
Berman et al., *New England Journal of Medicine*, vol. 363, no. 5, pp. 454–461, 2010.	Not systematic review
Vickers and Maschino, *Acupuncture in Medicine*, vol. 27, no. 3, pp. 126-127, 2009.	Not systematic review
Kelly, *American Family Physician*, vol. 80, no. 5, pp. 481–484, 2009.	Not systematic review
Rooney, *Internet Journal of Advanced Nursing Practice*, vol. 9, no. 2, p. 6p, 2008.	Not systematic review
Pennick and Young, *Cochrane Database of Systematic Reviews*, no. 2, Article ID CD001139, 2007.	No separate data of acupuncture for back pain
Luijsterburg et al., *European Spine Journal*, vol. 16, no. 7, pp. 881–899, 2007.	Unavailable data of acupuncture
Shen et al., *Journal of the American Academy of Orthopaedic Surgeons*, vol. 14, no. 8, pp. 477–487, 2006.	Not systematic review
Spearing et al., *APLAR Journal of Rheumatology*, vol. 8, no. 1, pp. 5–15, 2005.	Not systematic review
Luo and Luo, *Journal of Clinical Acupuncture and Moxibustion*, vol. 21, no. 6, pp. 10–14, 2005.	Insufficient data to judge study types
Liu, *Chinese Journal of Clinical Rehabilitation*, vol. 9, no. 18, pp. 195–197, 2005.	Not systematic review
Linde et al., *Forschende Komplementarmedizin und Klassische Naturheilkunde*, vol. 12, no. 4, pp. 225–227, 2005.	Commentary
Kluger and Bachmann, *Deutsche Zeitschrift fur Akupunktur*, vol. 48, no. 4, pp. 37–39, 2005.	Commentary
Furlan et al., *Spine*, vol. 30, no. 8, pp. 944–963, 2005.	Duplicated systematic reviews
Caroli et al., *Geriatric and Medical Intelligence*, vol. 14, no. 1, pp. 51–54, 2005.	Not systematic review
Maher, *Orthopedic Clinics of North America*, vol. 35, no. 1, pp. 57–64, 2004.	Not systematic review
Ernst, *Best Practice & Research: Clinical Rheumatology*, vol. 18, no. 4, pp. 539–556, 2004.	Not systematic review
Ngu et al., *Seminars in Spine Surgery*, vol. 15, no. 4, pp. 384–392, 2003.	Not systematic review
Hanada, *Best Practice & Research: Clinical Rheumatology, vol. 17, no. 1, pp. 151–166, 2003. *	Not systematic review
Eshkevari, *Journal of the American Association of Nurse Anesthetists*, vol. 71, no. 5, pp. 361–370, 2003.	Not systematic review
Abdulrazzaq et al., *Emirates Medical Journal*, vol. 21, no. 2, pp. 128–132, 2003.	Not systematic review
Young and Jewell, *Cochrane Database of Systematic Reviews*, no. 1, Article ID CD001139, 2002.	No separate data of acupuncture for back pain
He and Ding, *Chinese Journal of Clinical Rehabilitation*, vol. 6, no. 14, pp. 2034-2035, 2002.	Not systematic review
Smith-Fassler and Lopez-Bushnell, *Holistic Nursing Practice*, vol. 15, no. 3, pp. 35–44, 2001.	Not systematic review
Haigh, *Reviews in Clinical Gerontology*, vol. 11, no. 3, pp. 277–283, 2001.	Not systematic review
van Tulder et al., *Cochrane Database of Systematic Reviews*, no. 2, Article ID CD001351, 2000.	Updated Cochrane review available
Smith et al., *Pain*, vol. 86, no. 1-2, pp. 119–132, 2000.	Narrative systematic review
van Tulder and Irnich, *Forschende Komplementarmedizin*, vol. 6, no. 3, pp. 154–157, 1999.	Commentary
van Tulder et al., *Spine*, vol. 24, no. 11, pp. 1113–1123, 1999.	Duplicated systematic reviews
Strauss, *Chiropractic Journal of Australia*, vol. 29, no. 3, pp. 112–118, 1999.	Narrative systematic review
Longworth and McCarthy, *Acupuncture in Medicine*, vol. 16, no. 1, pp. 18–31, 1998.	Not systematic review
Ernst, *Fortschritte der Medizin*, vol. 116, no. 1-2, pp. 20–26, 1998.	Not systematic review
Longworth and McCarthy, *Journal of Alternative & Complementary Medicine*, vol. 3, no. 1, pp. 55–76, 1997.	Not systematic review
Birch et al., *Journal of Alternative & Complementary Medicine*, vol. 2, no. 1, pp. 101–124, 1996.	Not systematic review
Ernst and Fialka, *Fortschritte der Medizin*, vol. 111, no. 27, pp. 420–422, 1993.	Not systematic review
Tan et al., *Baillière's Clinical Rheumatology*, vol. 6, no. 3, pp. 629–655, 1992.	Not systematic review
Ceniceros, *Journal of Neurological and Orthopaedic Medicine and Surgery*, vol. 13, no. 4, pp. 263–266, 1992.	Not systematic review
Chen, *American Journal of Acupuncture*, vol. 18, no. 4, pp. 305–323, 1990.	Not systematic review

## Appendices

### A. Search Strategy

#### A.1. MEDLINE via Ovid Interface (from Inception to February 21, 2014)

1. review.pt.  (1834993)
2. meta analysis/(44357)
3. (systematic$ adj review$).tw  (51995)
4. (meta analy$).tw  (58477)
5. or/(1)–(4)  (1886676)
6. acupuncture/(1167)
7. exp acupuncture ear/(249)
8. exp acupuncture therapy/(16133)
9. exp acupuncture points/(3751)
10. needles/(10577)
11. electroacupuncture/(2424)
12. trigger points/(95)
13. moxibustion/(1068)
14. acupuncture$.tw  (14262)
15. needl$.tw  (84315)
16. (electro$ adj acupuncture$).tw  (603)
17. (auricul$ adj acupuncture$).tw  (241)
18. (warm$ adj acupuncture$).tw  (9)
19. (dry needl$).tw  (144)
20. (trigger-point$ adj therap$).tw  (60)
21. or/(6)–(20)  (104383)
22. low back pain/(13616)
23. back pain/(14399)
24. sciatica/(4067)
25. (low$ back pain$).tw  (17258)
26. (back pain$).tw  (28577)
27. backach$.tw  (2035)
28. lumbago$.tw  (1081)
29. dorsalgia$.tw  (66)
30. or/(22)–(29)  (44297)
31. (5) and (21) and (30)  (240)
32. animals/not humans/(3791961)
33. (31) not (32)  (237).

#### A.2. EMBASE via Ovid Interface (from Inception to February 21, 2014)

1. review.pt.  (2062617)
2. systematic review/(70772)
3. meta analysis/(80958)
4. (systematic$ adj review$).tw.  (67796)
5. (meta analy$).tw.  (79056)
6. or/(1)–(5)  (2157852)
7. acupuncture/or exp acupuncture needle/(29367)
8. needle/or exp acupuncture needle/(33506)
9. electroacupuncture/(4036)
10. trigger point/th(1)
11. moxibustion/(1534)
12. acupuncture$.tw.  (21814)
13. needl$.tw.  (121772)
14. (electro$ adj acupuncture$).tw.  (910)
15. (auricul$ adj acupuncture$).tw.  (378)
16. (warm$ adj acupuncture$).tw.  (12)
17. (dry needl$).tw.  (217)
18. (trigger point$ adj therap$).tw  (67)
19. or/(7)–(18)  (157198)
20. low back pain/rh, th  (7306)
21. backache/rh, th  (3422)
22. sciatica/or exp intervertebral disk hernia/or exp lumbar disk hernia/(20967)
23. (low$ back pain$).tw.  (24749)
24. (back pain$).tw.  (40978)
25. backach$.tw.  (3056)
26. lumbago$.tw.  (1922)
27. dorsalgia$.tw.  (132)
28. or/(20)–(27)  (66623)
29. (6) and (19) and (28)  (573)
30. animals/not humans/(1420149)
31. (29) not (30)  (570).

#### A.3. AMED via Ovid Interface (from Inception to February 21, 2014)

1. review.pt.  (6652)
2. exp Evidence based medicine/or Meta analysis/(2351)
3. (systematic$ adj review$).mp.  (2262)
4. meta analy$.mp.  (965)
5. or/(1)–(4)  (9978)
6. Acupuncture/or exp Acupuncture therapy/(9291)
7. exp Acupoints/or Needles/(1656)
8. Electroacupuncture/(744)
9. Ear acupuncture/(388)
10. Needling/(288)
11. Moxibustion/(475)
12. acupuncture$.mp.  (8895)
13. needl$.mp.  (1637)
14. (electro$ adj acupuncture$).mp.  (202)
15. (auricul$ adj acupuncture$).mp.  (115)
16. (warm$ adj acupuncture$).mp.  (11)
17. dry needl$.mp.  (42)
18. (trigger-point$ adj therap$).mp.  (31)
19. or/(6)–(18)  (10217)
20. Low back pain/(3791)
21. Backache/(1709)
22. Sciatica/(135)
23. low$ back pain$.mp.  (4895)
24. back pain$.mp.  (5808)
25. backach$.mp.  (1746)
26. lumbago$.mp.  (43)
27. dorsalgia$.mp.  (3)
28. or/(20)–(27)  (6642)
29. (5) and (19) and (28)  (42)
30. animals/not humans/(4282)
31. (29) not (30)  (42).

#### A.4. Cochrane Database of Systematic Reviews via Ovid Interface (from Inception to February 21, 2014)

1. systematic review.pt.  (5806)
2. (systematic$ adj review$).mp.  (6840)
3. meta analy$.mp.  (6828)
4. or/(1)–(3)  (8050)
5. acupuncture$.mp.  (398)
6. acupuncture therap$.mp.  (92)
7. acupuncture point$.mp.  (96)
8. acupuncture ear.mp.  (28)
9. needl$.mp.  (496)
10. electroacupuncture$.mp.  (78)
11. (auricul$ adj acupuncture$).mp.  (30)
12. (warm$ adj acupuncture$).mp.  (2)
13. (electro$ adj acupuncture$).mp.  (65)
14. dry needl$.mp.  (20)
15. (trigger-point$ adj therap$).mp.  (6)
16. trigger$ point$.mp.  (61)
17. moxibust$.mp.  (72)
18. or/(5)–(17)  (787)
19. low$ back pain$.mp.  (155)
20. back pain$.tw.  (318)
21. backach$.mp.  (88)
22. lumbago$.mp.  (49)
23. sciatica$.mp.  (64)
24. dorsalgia$.mp.  (35)
25. or/(19)–(24)  (356)
26. (4) and (18) and (25)  (87)
27. human$.mp.  (7049)
28. (26) and (27)  (82).

### B. Excluded Systematic Reviews

See Table 6.
